# Long-Term Spinal Cord Stimulation After Chronic Complete Spinal Cord Injury Enables Volitional Movement in the Absence of Stimulation

**DOI:** 10.3389/fnsys.2020.00035

**Published:** 2020-06-30

**Authors:** Isabela Peña Pino, Caleb Hoover, Shivani Venkatesh, Aliya Ahmadi, Dylan Sturtevant, Nick Patrick, David Freeman, Ann Parr, Uzma Samadani, David Balser, Andrei Krassioukov, Aaron Phillips, Theoden I. Netoff, David Darrow

**Affiliations:** ^1^Department of Biomedical Engineering, University of Minnesota, Minneapolis, MN, United States; ^2^Department of Neurosurgery, University of Minnesota, Minneapolis, MN, United States; ^3^Department of Bioinformatics and Computational Biology, University of Minnesota, Minneapolis, MN, United States; ^4^Division of Neurosurgery, VA Healthcare System, Minneapolis, MN, United States; ^5^International Collaboration on Repair Discoveries, Division of Physical Medicine and Rehabilitation, University of British Columbia, Vancouver, BC, Canada; ^6^Department of Physiology and Pharmacology, Cumming School of Medicine, University of Calgary, Calgary, AB, Canada; ^7^Division of Neurosurgery, Hennepin County Medical Center, Minneapolis, MN, United States

**Keywords:** spinal cord injury, spinal cord stimulation, volitional movement, traumatic spinal cord injury, neuromodulation, human

## Abstract

**Background:** Chronic spinal cord injury (SCI) portends a low probability of recovery, especially in the most severe subset of motor-complete injuries. Active spinal cord stimulation with or without intensive locomotor training has been reported to restore movement after traumatic SCI. Only three cases have been reported where participants developed restored volitional movement with active stimulation turned off after a period of chronic stimulation and only after intensive rehabilitation with locomotor training. It is unknown whether restoration of movement without stimulation is possible after stimulation alone.

**Objective:** We describe the development of spontaneous volitional movement (SVM) without active stimulation in a subset of participants in the Epidural Stimulation After Neurologic Damage (ESTAND) trial, in which locomotor training is not prescribed as part of the study protocol, and subject’s rehabilitation therapies are not modified.

**Methods:** Volitional movement was evaluated with the Brain Motor Control Assessment using sEMG recordings and visual examination at baseline and at follow-up visits with and without stimulation. Additional functional assessment with a motor-assisted bicycle exercise at follow-up with and without stimulation identified generated work with and without effort.

**Results:** The first seven participants had ASIA Impairment Scale (AIS) A or B thoracic SCI, a mean age of 42 years, and 7.7 years post-injury on average. Four patients developed evidence of sustained volitional movement, even in the absence of active stimulation after undergoing chronic epidural spinal cord stimulation (eSCS). Significant increases in volitional power were found between those observed to spontaneously move without stimulation and those unable (*p* < 0.0005). The likelihood of recovery of spontaneous volitional control was correlated with spasticity scores prior to the start of eSCS therapy (*p* = 0.048). Volitional power progressively improved over time (*p* = 0.016). Additionally, cycling was possible without stimulation (*p* < 0.005).

**Conclusion:** While some SVM after eSCS has been reported in the literature, this study demonstrates sustained restoration without active stimulation after long-term eSCS stimulation in chronic and complete SCI in a subset of participants. This finding supports previous studies suggesting that “complete” SCI is likely not as common as previously believed, if it exists at all in the absence of transection and that preserved pathways are substrates for eSCS-mediated recovery in clinically motor-complete SCI.

**Clinical Trial Registration:** www.ClinicalTrials.gov, identifier NCT03026816.

## Introduction

Almost 800,000 people suffer from traumatic spinal cord injury (SCI) worldwide every year (Kumar et al., 2018). While some recovery is expected after acute SCI, chronic SCI carries a stable prognosis with low probability of recovery. After the first year of injury, less than 2% of patients with motor complete spinal injury will become incomplete by the fifth year after injury (Kirshblum et al., 2004). Interventions for chronic complete SCI generally focus on the medical management of SCI-related complications, therapies to prevent musculoskeletal deterioration, and to provide adaptive strategies. Currently, predictors of neurological recovery in the acute phase include initial neurological status, incomplete injuries, and presence of a zone of partial preservation on imaging studies in complete injuries (Wilson et al., 2012). Only intensive neurorehabilitative therapies, such as body weight-supported treadmill training, have level 3 evidence for improving functional ambulation in chronic SCI, and this likelihood is greater in motor incomplete injuries (Lam et al., 2007).

Epidural spinal cord stimulation (eSCS, SCS, or estim) has long been used for the treatment of chronic pain (Shealy et al., 1967) and is originally based on Melzack and Wall’s (1965) gate control theory for peripheral neuromodulation of pain perception. As a neuromodulation platform capable of stimulating the central and peripheral nervous system, the therapeutic application of eSCS has been attempted on multiple fronts including Parkinson’s disease, MS, and SCI (Illis et al., 1980; Barolat et al., 1995; de Andrade et al., 2016). Several small clinical reports of spinal cord stimulation after chronic SCI have documented a promising potential to restore volitional movement in an immediate and long-term fashion (Angeli et al., 2018; Gill et al., 2018; Wagner et al., 2018). However, these improvements are achieved only within the context of intensive locomotor training coupled with eSCS.

The mechanisms by which electrical stimulation restores supraspinal control or modulates the function of the spinal cord remain unclear. Careful electrophysiology during motor-control tasks has revealed subtle supraspinal control in more than 80% of participants with clinically motor-complete injuries (Sherwood et al., 1992), which indicates the presence of clinically silent supraspinal tracts potentially amenable to electrical stimulation. Acutely, epidural electrical stimulation primarily activates monosynaptic reflexes and generates complex burst-like patterns initiated in the dorsal roots, identified as short-latency compound muscle action potentials (Minassian et al., 2004). At a minimum, by activating collateral dorsal root sensory projections, stimulation may modulate the excitability of local circuitry to allow for diminished and quiescent supraspinal activity to exert greater influence.

While the potential biological effects of *chronic* eSCS on the function of injured spinal cords remain unexplored, a few reports exist that highlight the possibility of the development of restored volitional movement even after eSCS is made inactive, usually after months of intensive rehabilitation and stimulation. The progressive development of improved function due to chronic neuromodulation would provide a potentially impactful therapeutic platform. However, it has not been reported without intensive rehabilitation, which requires significant additional cost and dedicated time (French et al., 2007).

The Epidural Stimulation After Neurologic Damage (ESTAND) trial tests the effect of eSCS after motor-complete thoracic SCI on volitional movement and autonomic function without implementing locomotor therapy (Darrow et al., 2019). After several participants unexpectedly began to exhibit clinical evidence of volitional movement *without* active eSCS, results were analyzed to further characterize this phenomenon. Here, we present preliminary analysis of the first seven patients in the trial across clinical observation, electrophysiology, and a functional bicycling task to characterize and compare those who did and did not develop volitional movement during periods without stimulation.

## Methods

### Subject Description

All the procedures described in this study were approved by the Hennepin Healthcare Research Institute Institutional Review Board and with an Investigational Device Exemption from the United States Food and Drug Administration. Patients with chronic, traumatic SCI (more than 1 year since injury) were recruited if they met the following criteria: older than 22 years of age, ASIA Impairment Scale (AIS) classification A or B with a neurological level of injury between C6 and T10, full arm and hand strength and intact segmental reflexes below the level of injury. Participants were excluded if they had medical or psychological comorbidities that would significantly increase the risk of surgery, severe dysautonomia (systolic blood pressure fluctuation below 50 or above 200 mmHg) during autonomic testing, contractures, pressure ulcers, recurrent urinary tract infection, unhealed spinal fracture, recent botulinum toxin use, or pregnancy. Once enrolled, subjects were asked to suspend any medications used for spasticity, for example, baclofen and oxybutynin. This analysis includes seven participants that have completed 80% or more of the study. The six participants that have completed the study in its entirety were enrolled for a range of 1.26–1.47 years. Overall, participants had a mean age (±SD) of 42 ± 11.4 years, and a mean time since injury (±SD) of 7.7 ± 4.8 years ranging from 3 to 17 years (Table 1). Three of the participants were female, and four were male. Utilizing the International Standards for Neurological Classification of Spinal Cord Injury (ISNCSCI) (Kirshblum et al., 2011), six participants were classified as AIS A, motor and sensory complete, and one subject was classified as AIS B, motor complete, and sensory incomplete. Subclinical motor complete injuries were further confirmed to be electrophysiologically complete with a baseline Brain Motor Control Assessment (BMCA) (Sherwood et al., 1996). All injuries were in the thoracic spine, with two participants at the T4 level, three participants at the T5 level, and two participants at the T8 level. Mechanisms of injury included falls, sports injuries, and motor vehicle accidents (MVA) (Table 1). Modified Ashworth Scale (MAS) (Meseguer-Henarejos et al., 2018) scores were collected at baseline before continuous stimulation therapy. A score of 0 to 4 was assigned to four muscle groups of each leg: hamstrings, quadriceps, gastrocnemius, and soleus. These scores were then averaged for a mean lower extremity MAS score. All subjects included in this manuscript completed all 13 follow-up visits, for approximately 1 year, except for subject 7, who had completed 8 follow-up visits or approximately 9 months in the study.

**TABLE 1:** Demographic information: *ages are rounded to the closest decade.

Subject	SVM	Age*	Years post injury	AIS	Lowest injury site	Vertebral fracture level	Mechanism of injury	Implant level
1	No	50	10.96	A	T8	T8	Fall	T11
2	Yes	30	8.21	A	T4	T4 &T5	Sports injury	T12
3	Yes	40	16.83	A	T8	T7 &T8	MVA	T12-L1
4	No	40	5.36	B	T5	T5 &T6	Fall	T11-T12
5	Yes	50	5.44	A	T5	T5	MVA	L1
6	Yes	60	4.02	A	T5	T4-T5 dislocation fracture	MVA	L1-L2
7	No	30	3.05	A	T4	T4	Sports injury	T12-L1

*Years post injury are set at the time of surgical implantation. Lowest site of injury is determined by the AIS neurological examination at screening. Spontaneous volitional movement (SVM): observed movement without stimulation. AIS, ASIA Impairment Scale; MVA, motor vehicle accident.*

### Imaging

**FIGURE 1:**
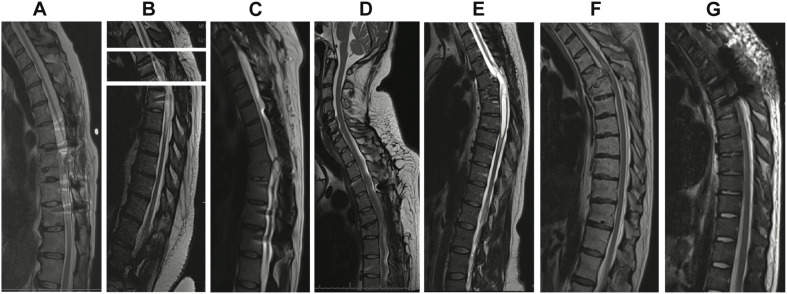
T2 sagittal thoracic magnetic resonance imaging (MRI) obtained prior to enrollment. **(A)** Subject 1: spinal cord changes include dorsal tethering at T7 and syrinx from T8 to T10. **(B)** Subject 2: syrinx at T7. Owing to coronal scoliosis, a single sagittal image did not provide a full view of the spinal canal. A composite image of three different slices with the midline of the central canal kept as the axis is shown. **(C)** Subject 3: T8 cord injury with cystic changes at the same level. **(D)** Subject 4: T5 cord injury. **(E)** Subject 5: T5 cord injury and syrinx extending from T5 to T7. **(F)** Subject 6: T5 cord injury. **(G)** Subject 7: T4 cord injury.

All participants provided thoracic spinal cord magnetic resonance imaging (MRI) at screening (Figure 1). Post-traumatic spinal cord changes observed on MRI included dorsal tethering, myelomalacia, syrinx, and cystic changes. In order to further categorize SCI severity and atrophy, spinal cord anteroposterior (AP) and transverse diameters were manually measured at C7–T1 (above the injury) and at T9 (below the injury) to assess for spinal cord atrophy (Freund et al., 2010). Both measures were matched to same-level normalized spinal cord diameters from healthy participants (Frostell et al., 2016) and their differences computed for statistical modeling. One subject’s MRI (Subject 7) was excluded from statistical analysis because it was obtained during the acute SCI period.

### Implantation and Follow-Up

**FIGURE 2:**
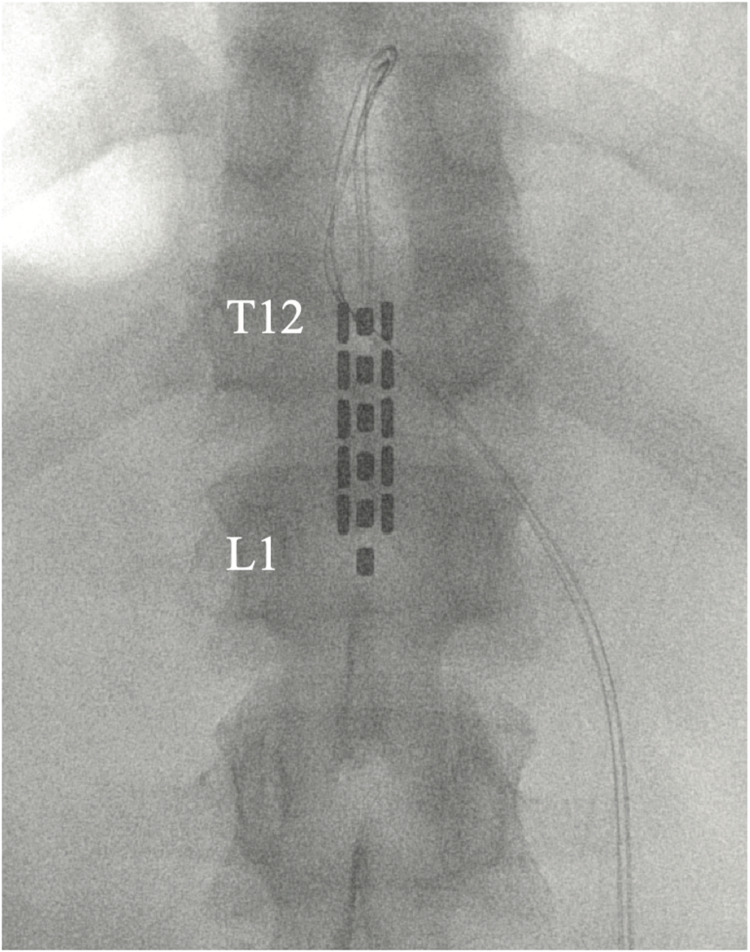
Placement of 5-6-5 epidural paddle lead through a T12 laminectomy, overlying the T12–L1 epidural space. Final lead placement is guided by intraoperative electromyogram (EMG).

All participants were implanted with an epidural stimulator upon enrollment after baseline data, including a detailed neurological exam and self-reported questionnaires, were collected. Participants underwent epidural placement of a three-column, 16-contact paddle lead through a T11–T12 laminectomy and subcutaneous placement of a primary cell internal pulse generator (IPG) (Tripole and Proclaim Elite, Abbott, Plano, TX, United States) in the lower lumbar area under general anesthesia (Figure 2). Intraoperative needle electromyogram (EMG) guided optimal paddle placement for symmetric and extended coverage of target spinal segments L2–S2. Starting 1 month after implantation, 13 follow-up assessments, 30–45 days apart, were conducted involving stimulator setting reprogramming and study assessments. Participants were provided with a patient programmer and allowed to utilize specific stimulation settings for different goals such as volitional movement, spasticity control, core strength, and autonomic functions. Participants could use the stimulation throughout each day (up to 24 h a day) after a month of gradual adjustment to time and amplitude. A detailed description of study methods can be found in a previous publication (Darrow et al., 2019). Observational data was collected using study personnel documentation from in-clinic follow-ups as well as subject self-reports.

### Brain Motor Control Assessment

The BMCA was conducted during subject screening and twice at each follow-up visit, without and with eSCS. The eSCS program was selected based on participants’ preferences during the previous month of eSCS use, as well as objective data on the current month’s settings (Darrow et al., 2019). The BMCA is a neurophysiological assessment of voluntary motor function using surface EMG over a series of three phases in the supine position: relaxation, reinforcement maneuvers, and voluntary movements (Sherwood et al., 1996). At the beginning of the trial, participants are instructed to follow a two-toned auditory cue as the marker for the beginning and end of each task. The reinforcement maneuvers include deep breath, neck flexion, Jendrassik maneuver, and bilateral shoulder shrug. The first set of voluntary movements includes hip and knee flexion/extension with both legs, and then isolated left and right sides. The second set of voluntary movements are ankle dorsiflexion and plantar flexion bilaterally, and the isolated left and right sides. The participants are asked to attempt the movements even if they are unable to do so and even if the requested movements are not produced. Surface EMG is measured through 15 pairs of surface electrodes on the following muscles bilaterally: paraspinal, iliopsoas, rectus femoris, tibialis anterior, extensor hallucis longus, gastrocnemius, rectus abdominis, and intercostals. A Nicollet EDX, ECR-16, research EMG is used at a sampling rate of 600 Hz.

Electromyogram was pre-processed by removing 60-Hz noise with a Fourier filter and then power calculated in windows of time by average root mean square (RMS) (Darrow et al., 2019). Command start and end times were marked with labeled event timestamps in the EMG acquisition system. Baseline time windows for each trial started 3 s before the auditory cue and ended 1 s before the auditory cue for all six volitional tasks. An auditory cue was manually synchronized with the event timestamp. Muscle activity power during volitional task time windows was averaged across muscle groups and across all tasks. Similarly, muscle activity voltage at rest before each task was averaged. The ratio of average muscle power during volitional tasks to baseline preceding the command to start volitional movement was used to measure strength of volitional control and is represented in decibels [dB; $10\,\log_{10}\left(\frac{P_{\text{v}}}{P_{\text{b}}}\right)$, where *P*_v_ is the power during volitional control, and *P*_b_ is the power in the immediately preceding window to the command to move] and will be referred to further on in this paper as volitional power.

### Stationary Bike

The Muvi 300 cycle from MOTOmed was used to assess functional movement capabilities. The Muvi 300 includes a motor-assisted setting that facilitates training with minimal muscle strength by switching from passive to active training without strain. The active assist motor can vary in speed (rpm) and resistance. When the force sensitivity threshold is met, the motor ceases and the patients pedal on their own in the bike’s active mode. The bicycle collects trial duration [seconds (s)], active and passive mode duration (s), active and passive distance traveled [meters (m)], active and passive speed [rotations per minute (rpm)], work done in active mode [kilojoules (kj)], and average and maximum energy produced during active phase [watts (W)].

The factorial design bicycling task was added after initial participants demonstrated enough movement capabilities with active eSCS to allow for more robust functional assessments during follow-up sessions. Therefore, the data capture window for each participant differs based on the relative enrollment date. If the bike was implemented after a subject initiated eSCS therapy, they did not undergo a baseline visit.

At the baseline visit, participants completed two motor-assisted bike trials: one passive trial where the subject was asked to relax and one active where the subject was asked to attempt to pedal. At each subsequent visit, the subject completed the same passive and active trials without stimulation and additional passive and active trials with selected preferred stimulation setting on. The duration of each trial was 2 min. As a result, there were two conditions without stimulation analyzed: (1) no effort, no stimulation and (2) maximal effort, no stimulation.

### Statistical Analysis

RStudio (2015) software was used for statistical analyses. Mann–Whitney U tests were used as non-parametric testing of differences between Spontaneous Volitional Movement (SVM) group and non-Spontaneous Volitional Movement (non-SVM) group. Generalized linear models were used to assess linear relationships for BMCA volitional power without stimulation during each study visit. Fixed effects tested to fit the model included: subjects, follow-up visit (time), Modified Ashworth scores before and after the study visit and volitional power with the stimulation on. A second model was used to assess the effects of spinal cord atrophy on average BMCA volitional power. Fixed effects tested to fit the model included: transverse and anteroposterior spinal cord diameter changes above and below the injury. When testing models with the same response variable, the best-fit model was chosen based on the lowest Akaike information criterion (AIC). Biking data included an excess of zero counts and overdispersed counts. It was therefore analyzed with a zero-inflated negative binomial regression that was found to be significantly superior to a negative binomial generalized linear model with the Vuong Non-nested Hypothesis test (*p* < 0.005). For all statistical analyses, a *p*-value of less than 0.05 was considered to be statistically significant. *p*-values are summarized in figures as one star (^∗^) for *p* < 0.05, two stars (^∗∗^) for *p* < 0.01, three stars (^∗∗∗^) for *p* < 0.001, and four stars (^****^) for *p* < 0.0001.

## Results

### Study Population

**FIGURE 3:**
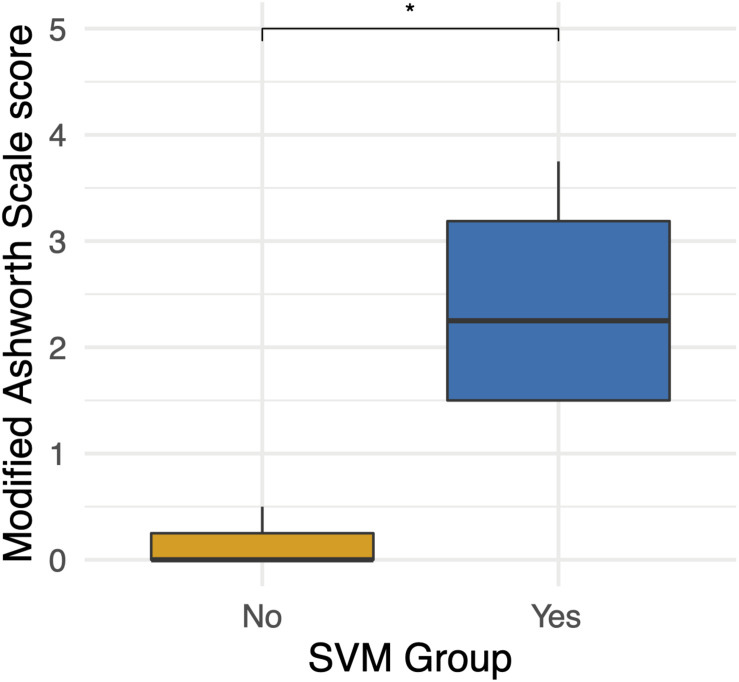
Differences in baseline Modified Ashworth Scale scores between the spontaneous volitional movement (SVM) group (Yes) and non-SVM group (No). Participants in the SVM group had significantly higher spasticity MAS scores than those in the non-SVM group. These differences were present before the start of continuous eSCS therapy. *p < 0.05.

Four participants unexpectedly developed sustained volitional movement with stimulation turned off, referred to as the SVM group. In order to ascertain group differences, participants from the SVM group were compared to the participants who only demonstrated movement with stimulation, referred to as the non-SVM group (*n* = 3) (Supplementary Table S1). Age *(p* = 0.285) and years post-injury *(p* = 0.476) were not significantly different between the SVM and non-SVM groups. The differences of anteroposterior (*p* = 0.114) and transverse (*p* = 0.212) spinal cord diameters from normal above the injury were not significantly different between SVM and non-SVM groups. The differences of anteroposterior (*p* = 0.4) and transverse (*p* = 0.4) spinal cord diameters from normal below the injury were not significantly different between SVM and non-SVM groups. Baseline spasticity scores (MAS) prior to continuous stimulation therapy were significantly higher at baseline in the SVM group (mean 2.44 ± 1.12) than in the non-SVM group (mean 0.17 ± 0.29; *p* = 0.048). This difference is depicted in Figure 3.

Across all participants, average daily stimulation use ranged from 5 to 21 h/day, with a mean of 13.7 ± 5.8 h/day. Total eSCS time at their final follow-up visit ranged from 101.2 to 454.5 days, with a mean of 255.3 ± 115.3 days. There were no statistically significant differences in the total amount of stimulation used (*p* = 0.629) nor average daily stimulation (*p* = 1) between SVM and non-SVM groups. Within the SVM group, participants had undergone a range of 67.1–244.6 total days of stimulation at the time of their first observed movement, with a median of 87 days.

While this study does not involve an intensive rehabilitative therapy component, study personnel collected self-reported information in order to characterize modalities and hours of therapy undergone before and during the study. Six participants reported receiving rehabilitative therapy during acute care as well as general and specialized in-patient rehabilitation. Prior to the study, 57% (4/7) of the participants reported receiving specialized SCI out-patient therapy, 29% (2/7) reported receiving general out-patient therapy, and 43% (3/7) completed therapy at home. After implantation of the epidural stimulator, 43% (3/7) of the participants reported receiving specialized SCI out-patient therapy, 14% (1/7) reported receiving general out-patient therapy, and 43% (3/7) completed therapy at home. Out of the six participants who responded to retrospective surveys, three reported changing their exercise routine post-implantation.

Participants in the SVM group practiced a wide range of exercises spanning from range of motion, aerobic exercises, and general upper- and lower-body strength exercises up to specialized SCI out-patient therapy through adaptive gyms, clinic services with a physical therapist, and even activity-based locomotor exercise programs for core strength, leg strength, and balance. participants from the non-SVM group engaged in a similar range of exercise modalities. Of note, one subject in the non-SVM group participated in rehabilitative therapy that included using a standing frame, exoskeleton, and functional electrical stimulation to aid with stretching sessions. Intensity of rehabilitation therapy varied throughout the overall subject population (Table 2).

**TABLE 2:** Weekly exercise schedule: reported exercises during study enrollment.

Subject	SVM	Exercises
1	No	Stretching and range of motion – 7× week, 30–45 min
2	Yes	Activity based locomotor exercise – 3× week, 60 min
3	Yes	Upper body exercises – 3× week, 60 min Leg exercises – 3× week, 20 min
4	No	Adaptive gym
5	Yes	Range of motion, strength training, aerobic exercise, in clinic rehabilitation with a physical therapist – 1× week, 120 min
6	Yes	Adaptive gym – 1–2× week, 60 min
7	No	Standing frame – 3–4× week, 40 min Exoskeleton – 1× week, 40 min Stretch sessions with electrodes – 2–3× week, 60 min

*Subjects are assigned to SVM or non-SVM groups based on observational data.*

### Observational Data

**FIGURE 4:**
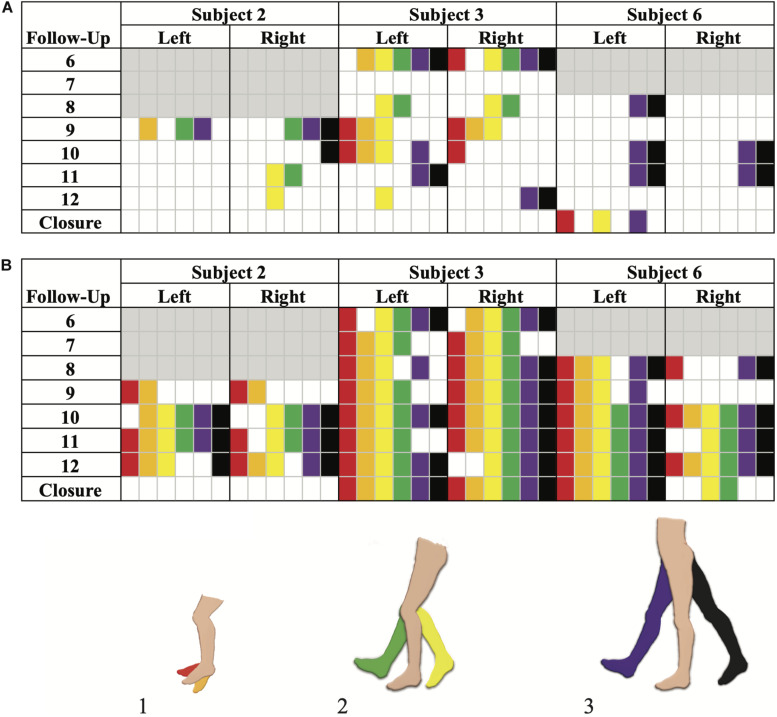
Brain Motor Control Assessment (BMCA) documented muscle activation without stimulation **(A)** and with stimulation **(B)** in the SVM group. Joint movements are color coded as follows: (1) red, ankle dorsiflexion and orange, ankle plantarflexion. (2) Yellow, knee flexion and green, knee extension. (3) Purple, hip flexion and black, hip extension. Follow-up visits 1–5 and Subject 5 are not included as the BMCA CRF had not been implemented yet. Each subject’s introduction of the CRF form is color-coded by gray boxes. Movements observed prior to the implementation of the BMCA CRF have not been included here. Recorded movements only occurred during the volitional task windows of the BMCA **(A)**. Recorded movements during BMCA in the *absence of stimulation*. Subject 3 demonstrated persistent movement in the absence of stimulation the earliest and across the most muscle groups among the SVM group. **(B)** Recorded movements during BMCA *with stimulation on* are included in order to exemplify how movement with stimulation is more prevalent earlier on in the study and across more muscle groups than movement without stimulation.

Volitional movement without epidural stimulation was observed among the four participants (two females, two males) in the SVM group as early as 3 months post-implantation. In three of the four participants, study personnel cited volitional movement without eSCS during the BMCA. A case report form (CRF) was implemented to document and characterize observed movements with and without stimulation (Supplementary Video S1). Of those three participants, 100% demonstrated hip flexion and extension as well as knee flexion without eSCS during at least one follow-up visit. Two out of the three exhibited knee extension as well as ankle dorsiflexion and plantar flexion during at least one follow-up visit (Figure 4A).

Subject 5 provided a self-report noting right hip adduction, knee flexion/extension, and plantar extension without eSCS at-home during month 13 (Supplementary Video S2). Volitional movement without eSCS was not observed during their in-clinic BMCA testing.

By comparison, movement with stimulation (Figure 4B) was observed to be more consistent across muscle groups and more prominent in range of motion than without stimulation. When stimulation was on, all participants from both the SVM and non-SVM groups achieved volitional movement to varying degrees of magnitude and extent. These results are observed early on in each subject’s study enrollment time, and they can be observed as a direct effect of acute stimulation.

### Brain Motor Control Assessment

**FIGURE 5:**
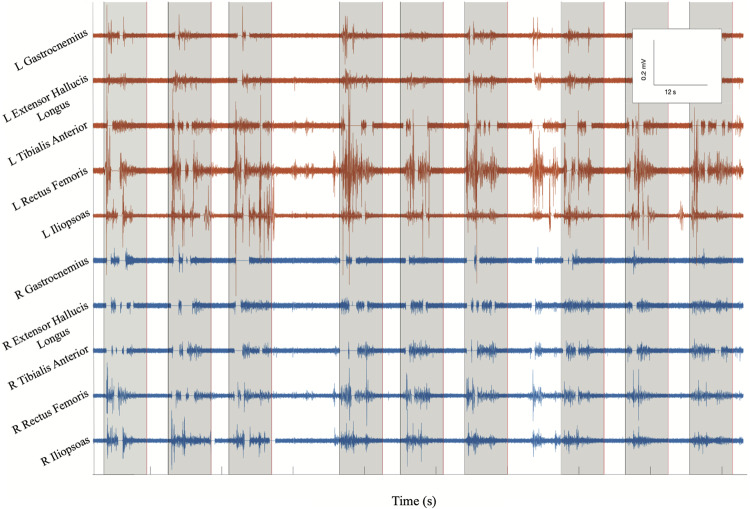
BMCA at Subject 2’s follow-up visit 13: surface EMG electrical activity recorded in volts over time for eight bilateral lower extremity muscle groups. Orange traces include left muscle groups and blue traces include right muscle groups. Gray boxes indicate volitional task events signaled by auditory cues. This subsample of EMG recording includes three cues for bilateral hip flexion, right hip flexion, and left hip flexion, respectively. EMG bursts can be seen to be synchronized with the auditory cue followed by silent periods at rest, demonstrating volitional activity.

**FIGURE 6:**
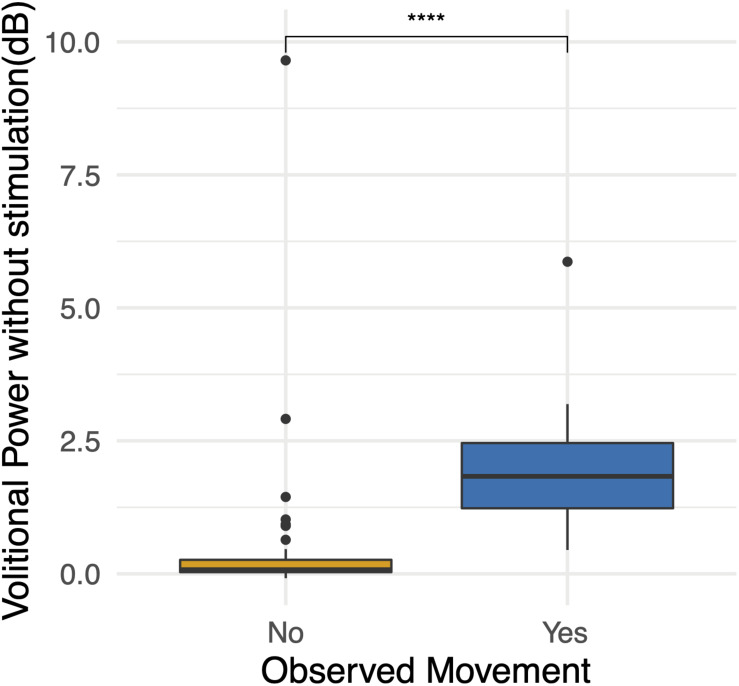
Observations during BMCA. Differences in volitional power without stimulation (dB) when movement was observed and recorded on case report forms. *High volitional power outliers when movement was not observed demonstrate the lower sensitivity of researcher observations.* For reference, volitional power of 3 dB represents an increase in muscle activity during volitional tasks of two times (200%) that of muscle activity at baseline. Volitional power of 10 represents an increase in muscle activity during volitional tasks of 10 times (1000%) that of the muscle activity at baseline. ***p < 0.001.

In order to characterize study observations, analysis of electrophysiological data from BMCA sessions without stimulation was performed. Sessions in which movement without stimulation was documented demonstrated increases in muscle activity specifically during volitional motor tasks compared to rest during baseline (Figure 5). The magnitude of volitional motor control is represented by volitional power, as this ratio corrects for any involuntary muscle activity at baseline related to spasticity or spasms. There was a significant difference in volitional power, controlling for involuntary movement at rest, when movement was recorded in BMCA CRFs (*p* < 0.0005) (Figure 6), meaning that clinical recognition of volitional movement is in agreement with EMG activity measured during the BMCA.

A generalized linear model was used to identify the effects of several variables on volitional power (dB) during volitional tasks without stimulation. Longitudinal explanatory variables tested included individual subject, follow-up visit, Modified Ashworth Scores during follow-up visits, and volitional power (dB) with stimulation on. When holding all other variables constant, there was a significant but weak effect of time (*p* = 0.016), a significant and strong positive effect from Subject 3 (*p* = 0.0005) who demonstrated the most significant improvement, a significant and moderate positive effect of spasticity before BMCA testing (*p* = 0.002) and a weak and trending toward significance positive effect of volitional power when stimulation was on (*p* = 0.066). To assess for SCI differences, a generalized linear model for average volitional power for each subject was tested with the difference between transverse and anteroposterior spinal cord diameters to normal spinal cord measures *above the injury* (C7, T1) as explanatory variables; there were no significant effects. In a generalized linear model for average volitional power for each subject with differences between transverse and anteroposterior spinal cord diameters to normal spinal cord measures *below the injury (T9)* as explanatory variables, there was a negative strong effect of the AP spinal cord diameter difference that trended toward significance (*p* = 0.071). In other words, greater anteroposterior spinal cord atrophy had a negative effect on the amount of volitional power achieved.

**FIGURE 7:**
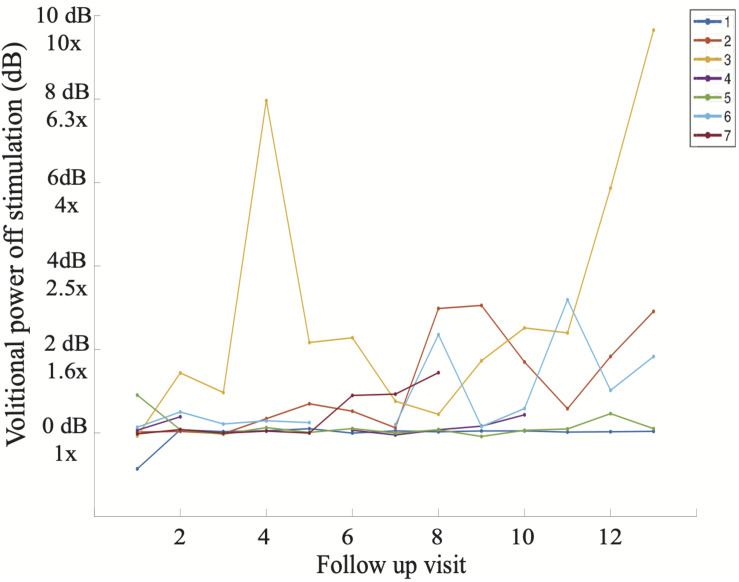
Average volitional power without stimulation (dB) at each study follow-up visit including all seven participants. It is apparent that Subject 3 demonstrated volitional movement the earliest in the study (follow-up visit 4) and with the greatest magnitude on sEMG, reaching volitional muscle activity 10 times greater (1000%) than that at baseline on follow-up visit 13.

**FIGURE 8:**
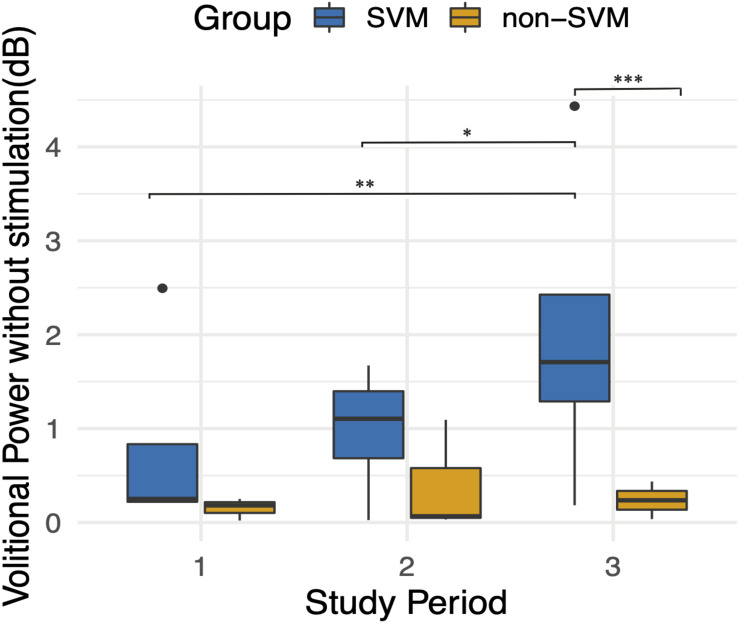
Average volitional power without stimulation (dB) during three follow-up study periods. Study period 1 included follow-up visits 1–5. Study period 2 included follow-up visits 6–9. Study period 3 included follow-up visits 10–13. An improvement over time is apparent only in the SVM group and is statistically significant between study periods 1 and 3 (*p* = 0.008) and between study periods 2 and 3 (*p* = 0.03). Volitional power between SVM and non-SVM groups is significantly greater in study period 3 (*p* < 0.001). *p < 0.05, **p < 0.01, ***p < 0.001.

When assessing for the independent effect of time, a trend is apparent as participants progress in follow-up visits (Figure 7), and the effect of time is significant when correcting for other fixed effects, as is mentioned above. With the exception of Subject 3’s early volitional activity at follow-up visit 4, the rest of the subject’s volitional power emerges later on in the study. While Subject 5 is included in the SVM group due to observed volitional movement outside of study assessments, these results are not reflected as volitional power that is apparent above baseline. On the other hand, while Subject 7 is included in the non-SVM group, they have not completed the study at the time of this manuscript submission, and the latest follow-up visits demonstrate a rise in volitional power above baseline. To correct for intersubject and between-visit variability, pooling observations between SVM and non-SVM groups during three different stages of the study allows for a clearer interpretation of the effect over time (Figure 8). Study periods were divided as follows: study period 1 corresponds to visits 1–5 (approximately 5 months), study period 2 corresponds to visits 6–9 (approximately 4 months), study period corresponds to visits 10–13 (approximately 4 months). In study period 2, greater volitional power in the SVM group than in the non-SVM group trends toward significance (*p* = 0.087), and in study period 3, there is significantly greater volitional power in the SVM group (*p* < 0.001). Furthermore, only within the SVM group is there a significant increase in volitional power from study period 1 to study period 2 (*p* = 0.008) and from study period 2 to study period 3 (*p* = 0.03).

### Bike

Stationary bicycle trials proved complementary to electrophysiological measures as a functional assessment. During one participant’s best bike trial, Subject 3 (Supplementary Video S3), they exerted 235 J of work that amounted to active pedaling without motor assistance for 94.2% of the trial time and 96.4% of the distance traveled. These results occurred after 6 months of chronic eSCS. In the SVM group, the earliest activity emerged at follow-up visit 6 and the latest at follow-up visit 10.

**FIGURE 9:**
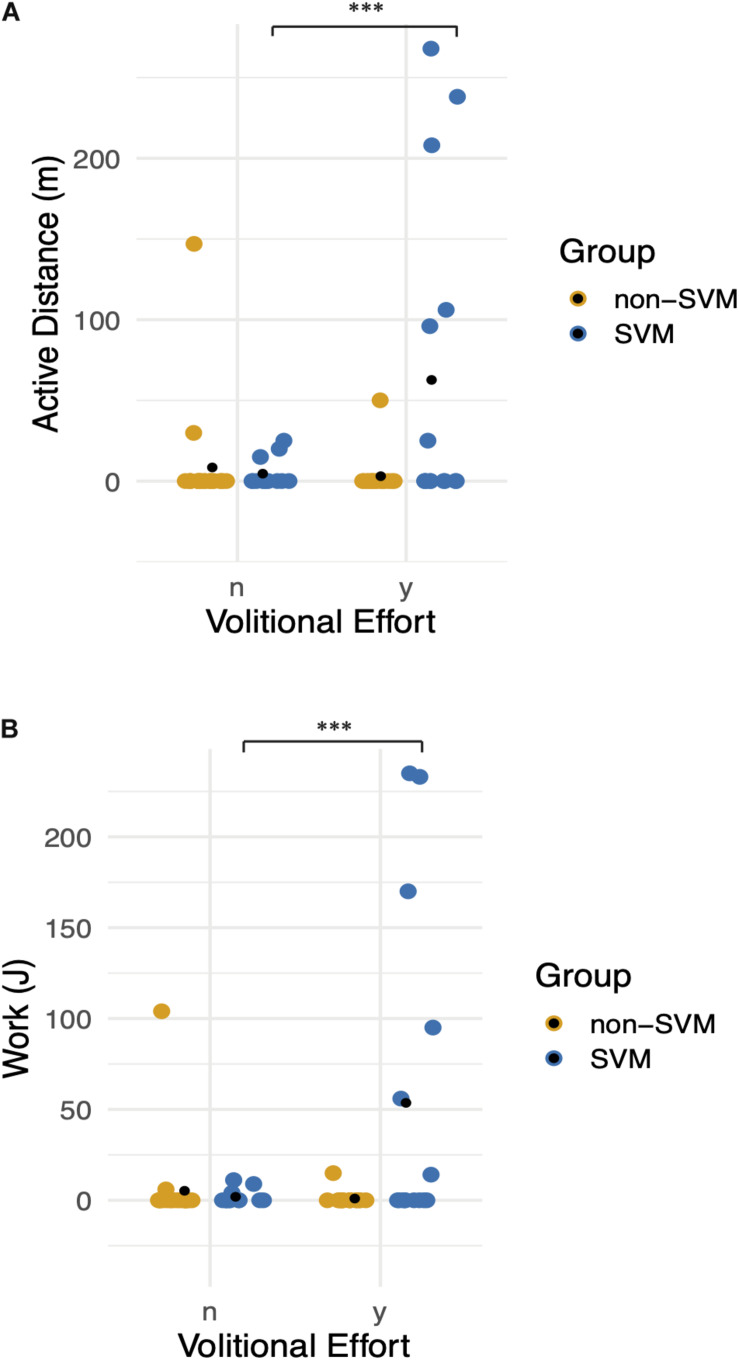
Functional movement without stimulation: graphs demonstrate differences between Groups (0: non-SVM group and 1: SVM group) and between volitional effort (*n* = no effort, *y* = with effort). Means are symbolized by black points. **(A)** Distance traveled without assistance of the bicycle motor is plotted, when correcting for zero inflated data. There is a significant positive effect on distance when participants from the SVM group attempted to pedal. **(B)** Pedaling work exerted is plotted, when correcting for zero inflated data. Participants in the SVM group significantly achieved greater work when they provided effort compared to the non-SVM group. *** refers to a *p*-value of <0.001.

A zero-inflated negative binomial regression for two selected dependent variables, distance traveled and work, was constructed including the following independent variables: individual participants and the interaction between groups (SVM vs. non-SVM) and pedaling effort provided. When holding all other variables constant, there was a significantly strong positive effect on distance traveled (*p* < 0.005) and work (*p* < 0.005) only when participants from the SVM group attempted to pedal (Figure 9).

## Discussion

Preliminary data from the ESTAND trial suggests that long-term or chronic eSCS can induce plastic changes in chronic, severely injured spinal cords through restored volitional movement without stimulation and without significant intensive rehabilitation. More than half of the first seven patients were observed to exhibit volitional movement without stimulation, which agreed with the more sensitive electrophysiology, and resulted in marked improvements in a functional cycling task.

None of the participants included in this cohort demonstrated traditional signs of discomplete spinal cord injuries before eSCS therapy began (Sherwood et al., 1992). Despite the fact that all participants exhibited motor-complete traumatic spinal cord injuries confirmed with MRI, clinical testing, and electrophysiological testing at screening, more than half of the participants demonstrated the reported improvements in volitional movement capabilities with stimulation *inactive*. It is important to emphasize that before these improvements were apparent, there was no indication that study participants had different responses to long-term eSCS because, grossly, all participants demonstrated improvements in volitional muscle activity when epidural stimulation was *active*. When comparing researcher-observed movements during BMCA off and on stimulation, active stimulation allowed for volitional joint movements that spanned across more muscle groups and more consistently across study visits. However, joint movements observed off stimulation represented a subset of those facilitated by eSCS at similar time points. This finding might indicate that some of the same circuits that are potentiated by active stimulation are those responsive to chronic eSCS-facilitated plasticity.

### Predictors of Recovery

In an effort to characterize each subject’s propensity for this type of recovery, several descriptive subject characteristics were included such as time since injury, mechanism of injury, injury level, vertebral fracture level, fusion level, and imaging studies. None of these factors were significant in determining if a subject would develop movement without stimulation. Overall, the heterogeneity between participants exemplifies how within the most severe subgroup of the AIS scale, there are no current adequate measures to characterize the functional capacity of the spinal cord. Adequate measures that reflect the degree of preserved and quiescent supraspinal tracts across the spinal cord lesion could allow for phenotyping those responsive to neuromodulation.

In our cohort, spasticity scores before initiating eSCS therapy were slightly greater in the SVM group than in the non-SVM group, which was statistically significant. Moreover, longitudinal spasticity scores before each BMCA session had a significant positive effect on volitional power. When assessing differences among motor-complete SCI participants, Sangari et al. (2019), compared spastic and non-spastic subgroups and reported that motor evoked potentials (MEP) were only present in the spastic subgroup, suggesting that spasticity might be a marker for preserved corticospinal tract axons (Sangari et al., 2019). Greater spasticity at baseline and during chronic eSCS therapy might reflect preserved but silent corticospinal tracts that served as substrates for the plastic effects of eSCS neuromodulation in the SVM group or may highlight the role that higher baseline spasticity may play in electrophysiology. Furthermore, high spasticity phenotypes might be more susceptible to the immediate depolarizing effects and long-term neuromodulation effects of eSCS that restore inhibition of uncontrolled spinal cord excitability and potentiate functional activation of the spinal cord (D’Amico et al., 2014). Although the mechanisms might be unclear, spasticity should be further assessed as a predictor and biomarker for eSCS-mediated spontaneous recovery.

Measures of spinal cord atrophy were also assessed as a marker of injury severity. Only anteroposterior atrophy below the level of injury was found to have a strong effect on volitional power that did not quite meet our criteria for significance. In chronic motor complete spinal cord injuries, Sangari et al. (2019), reported MEP size to be positively correlated with the degree of spared tissue in lateral regions of the spinal cord above the injury. Our results should be interpreted with caution as spinal cord diameter changes have been reported as a measure of SCI severity only *above* the level of injury (Freund et al., 2010; Sangari et al., 2019), and the measurements in this cohort were limited by suboptimal MRI studies due to hardware artifacts and variability in SCI chronicity. As a result, imaging metrics did not prove to be useful predictors of recovery into the SVM group.

Most importantly, despite the large number of standard characteristics used to describe SCI, all but spasticity proved to be ineffective at predicting the variability in the development of movement without stimulation despite a uniform improvement with active stimulation. In other words, there are no major predictors of the development of movement without stimulation identified thus far that would restrict the potential future use of eSCS as a therapy.

### Role of Concurrent Rehabilitation in eSCS Recovery

Since the first case of eSCS in SCI aiming to restore volitional movement was reported in 2004 (Carhart et al., 2004), there have been reports of three chronic SCI patients with eSCS therapy who have regained some level of volitional movement in the absence of stimulation after intensive locomotor therapy. Angeli et al. (2014), reported movement without eSCS in one 32-year-old male patient with an AIS B injury after 38 weeks of intensive locomotor training that included 80 sessions of full weight-bearing stand training and 80 sessions of step training with body weight support as well as home-based volitional training (Rejc et al., 2017). Wagner et al. (2018), also reported two participants, a 28-year-old male with AIS C injury and a 35-year-old male with AIS D injury both enrolled 6 years after injury, who demonstrated improvements in walking indices and motor scores without eSCS after participating in 5 months post-implantation of overground and treadmill locomotor training using a gravity assist device four to five times a week. In contrast, the ESTAND study did not prescribe the use of intensive locomotor training. In addition to their baseline rehabilitation therapies (described in Table 2), participation in this study involved a minimum of 10 min of daily visually cued flexion–extension tasks at home, 90 min of supine flexion–extension tasks during monthly BMCA, and 10 min of motor-assisted pedaling during monthly bike testing. Participants utilized eSCS in their daily activities according to their needs and preferences up to 24 h per day, and more independent rehabilitation therapy was not associated with any clear benefit.

Despite heterogeneity in daily time and effort dedicated to physical therapy among all participants, more than half of the participants (SVM group) demonstrated progressive statistically significant improvements in volitional movement in the off stimulation state during the study period. As a result, there were statistically significant improvements in the ability to cycle without assistance, providing the basis for cost-effective home-based therapies to provide incremental improvements in muscle mass, cardio-metabolic risk factors and activities of daily living as well as a platform for activity-dependent plasticity (Shen et al., 2018; Gorgey et al., 2019).

While activity-based plasticity is often associated with rehabilitation therapies, there is a possibility that directly increasing volitional movement, increased use, and reliance on these improvements may facilitate more subtle and chronic activity-based plasticity in the sense that directed motor control during normal everyday life drives plastic changes. However, more intensive concurrent rehabilitation outside of the study did not drive further recovery, which is exemplified by the fact that the participant who underwent the most extensive specialized SCI therapy before and during the study did not develop spontaneous movement without stimulation. To our knowledge, this is the first report of eSCS-induced plasticity of volitional movement in the absence of concurrent prescribed intensive locomotor training therapy after motor-complete SCI. While the E-STAND trial remains generalizable by allowing for a wide range of independent therapy, careful data collection of previous therapy regimens may prove useful for assessing further contribution through modeling.

### Limitations and Future Directions

One limitation in this study relates to an undefined off-stimulation time period. As our protocol allows participants to use as much stimulation as they require for their daily activities and comfort. As such, there was no established eSCS-weaning time beyond a minimum of 2 h when testing for off-stimulation activity. An important confounder to consider is whether the reported results might be related to stimulation carry-over effect, described as temporarily persisting changes in spinal cord circuit excitability. This has been described clinically in relation to spasticity modulation in SCI patients as lasting from hours to days (Cook, 1976; Dimitrijevic M. M. et al., 1986; Dimitrijevic M. R. et al., 1986; Barolat et al., 1988). Whether the effects in the absence of eSCS in the participants in this study are temporary or persistent over longer periods of time with stimulation off will have to be further assessed.

In this manuscript, the observed movements during the BMCA were not matched to the intended volitional task. Instead, pooling of observed movements was compared to pooled EMG muscle activation during all volitional tasks. In the future, a thorough analysis of the accuracy of muscle activation for each intended joint movement should be performed. The preliminary results at this stage were not sufficiently powered to assess these outcomes. Here we demonstrate the robust evidence of muscle activity magnitude. Muscle activity accuracy will have to be assessed in future larger studies.

Although this is the largest group reported of SCI patients treated with eSCS to restore volitional movement, results should be interpreted with caution due to the small number of participants. In the future, studies with larger cohorts might allow for adequate eSCS therapy phenotyping. In-depth analysis of stimulation usage might point to a dose–response relationship that was not apparent in this study. Furthermore, neurophysiological testing such as somatosensory evoked potentials, electroencephalogram, and transcranial magnetic stimulation motor-evoked potentials might allow for categorizing the effects of chronic eSCS on ascending pathways, cortical representation, and descending pathways, respectively. Correlating these results with high-resolution MRI at enrollment to detect spinal cord area differences might aid in further characterizing the heterogeneity of spinal cord injuries and identifying the degree of preserved pathways that may serve as substrates for recovery (Freund et al., 2010). With these results, we hope to further evidence the role of eSCS in SCI rehabilitation and exemplify how the effects of *chronic* eSCS are only starting to be apparent.

## Data Availability Statement

The datasets presented in this article are not readily available because the ESTAND trial is currently ongoing and datasets retain some identifiable information. A limited dataset may be made available. Requests to access the datasets should be directed to David P. Darrow, darro015@umn.edu.

## Ethics Statement

The studies involving human participants were reviewed and approved by the Human Subjects Research Committee, Hennepin Healthcare System. The patients/participants provided their written informed consent to participate in this study. Written informed consent was obtained from the individuals for the publication of any potentially identifiable images or data included in this article.

## Author Contributions

DD provided all oversight for the study. DD, APa, AK, APh, US, and TN designed the study. DD, DF, APa, and US performed the critical surgical procedures of this study. CH, AA, SV, DS, DB, and DF performed the study procedures and data collection. DD, TN, NP, and IP analyzed the data. IP wrote the manuscript with support from DD, CH, AA, SV, and DS. All authors provided critical feedback and helped shape the research, analysis, and manuscript, and approved the final manuscript.

## Conflict of Interest

US reports having no conflicts of interest relevant to this article. DD, AP, and TN report having several patents related to neuromodulation and are cofounders of a neuromodulation company. The remaining authors declare that the research was conducted in the absence of any commercial or financial relationships that could be construed as a potential conflict of interest.

## Supplementary Material

TABLE S1 Summary of means and standard deviations *(SD)* for all variables used to assess differences between groups. *P*-values are obtained from single variable Mann-Whitney U tests. SVM: Spontaneous volitional movement. Non-SVM: No spontaneous volitional movement.

VIDEO S1 BMCA video recording of subject 3 with stimulation off. Subjects lie supine with surface electromyography electrodes placed while they perform volitional tasks triggered by auditory cues. At follow up 6, the subject achieves bilateral but predominantly left hip internal rotation and flexion as well as left dorsiflexion of the toes. At follow up 9, he again achieves left hip internal rotation and flexion as well as left dorsiflexion of the toes.

VIDEO S2 Home video recording of subject 4 demonstrates volitional right knee extension in the absence of stimulation.

VIDEO S3 Stationary bike video recording of subject 3 at Follow-up visit 6. This trial involves 2 min when subjects intend to pedal with stimulation off. In this particular trial shown, the subject achieved active pedaling for 113 seconds (94% of trial time) for a distance of 268 meters (96% of trial distance).

## Abbreviations

BMCA: Brain Motor Control Assessment
eSCS: epidural spinal cord stimulation
ESTAND: Epidural Stimulation After Neurologic Damage
MAS: Modified Ashworth Scale
SVM: Spontaneous Volitional Movement.
