# Supplementary material for: Long-Term Spinal Cord Stimulation After Chronic Complete Spinal Cord Injury Enables Volitional Movement in the Absence of Stimulation
Source: Front Syst Neurosci. 2020 Jun 30;14:35. doi: 10.3389/fnsys.2020.00035 (PMC7340010; doi:10.3389/fnsys.2020.00035)
Supplement: TABLE S1 — Summary of means and standard deviations (SD) for all variables used to assess differences between groups. P-values are obtained from single variable Mann-Whitney U tests. SVM: Spontaneous volitional movement. Non-SVM: No spontaneous volitional movement. [file Table_1.docx]

Supplementary Table 1. Summary of means and standard deviations (SD) for all variables used to assess differences between groups. P-values are obtained from single variable Mann-Whitney U tests. SVM: Spontaneous volitional movement. Non-SVM: No spontaneous volitional movement.

| Variable | | SVM group (n=4) | non-SVM group (n=3) |
| --- | --- | --- | --- |
| Age (years) | Mean | 49.25 | 38.00 |
|  | SD | 7.63 | 13.11 |
|  | p-value | 0.285 | |
| Years Post-injury (years) | Mean | 9.31 | 6.46 |
|  | SD | 5.84 | 4.07 |
|  | p-value | 0.426 | |
| Anteroposterior spinal cord differences from normal above the injury (mm) | Mean | -2.11 | -1.18 |
|  | SD | 0.81 | 0.42 |
|  | p-value | 0.114 | |
| Transverse spinal cord differences from normal above the injury (mm) | Mean | -2.33 | -0.77 |
|  | SD | 0.65 | 1.44 |
|  | p-value | 0.212 | |
| Anteroposterior spinal cord differences from normal below the injury (mm) | Mean | -0.63 | -0.37 |
|  | SD | 0.95 | 0.20 |
|  | p-value | 0.4 | |
| Transverse spinal cord differences from normal below the injury (mm) | Mean | -1.28 | -0.70 |
|  | SD | 0.70 | 0.98 |
|  | p-value | 0.4 | |
| MAS baseline score | Mean | 2.44 | 0.17 |
|  | SD | 1.12 | 0.29 |
|  | p-value | 0.048 | |
| Total stimulation used (hours) | Mean | 239.35 | 276.63 |
|  | SD | 156.56 | 42.57 |
|  | p-value | 0.629 | |
| Average daily stimulation (hours per day) | Mean | 13.46 | 14.00 |
|  | SD | 8.07 | 1.90 |
|  | p-value | 1 | |
